# Differences in the link between social trait judgment and socio-emotional experience in neurotypical and autistic individuals

**DOI:** 10.1038/s41598-024-56005-5

**Published:** 2024-03-05

**Authors:** Shangcheng Zhao, Runnan Cao, Chujun Lin, Shuo Wang, Hongbo Yu

**Affiliations:** 1 Department of Psychological and Brain Sciences, University of California Santa Barbara, Santa Barbara, CA 93106 USA; 2 Department of Radiology, Washington University in St. Louis, St. Louis, MO 63110 USA; 3 Department of Psychology, University of California San Diego, San Diego, CA 92093 USA

**Keywords:** Social trait perception, Interpersonal transgression, Responsibility, Guilt, Human behaviour, Social behaviour

## Abstract

Neurotypical (NT) individuals and individuals with autism spectrum disorder (ASD) make different judgments of social traits from others’ faces; they also exhibit different social emotional responses in social interactions. A common hypothesis is that the differences in face perception in ASD compared with NT is related to distinct social behaviors. To test this hypothesis, we combined a face trait judgment task with a novel interpersonal transgression task that induces measures social emotions and behaviors. ASD and neurotypical participants viewed a large set of naturalistic facial stimuli while judging them on a comprehensive set of social traits (e.g., warm, charismatic, critical). They also completed an interpersonal transgression task where their responsibility in causing an unpleasant outcome to a social partner was manipulated. The purpose of the latter task was to measure participants’ emotional (e.g., guilt) and behavioral (e.g., compensation) responses to interpersonal transgression. We found that, compared with neurotypical participants, ASD participants’ self-reported guilt and compensation tendency was less sensitive to our responsibility manipulation. Importantly, ASD participants and neurotypical participants showed distinct associations between self-reported guilt and judgments of criticalness from others' faces. These findings reveal a novel link between perception of social traits and social emotional responses in ASD.

## Introduction

Smooth social interactions, which depend on having intact social cognitive and affective processes, are crucial for our survival and well-being^1^. People with autism spectrum disorder (ASD) often find it difficult to navigate the social world due to impairments in key social cognitive and affective processes, such as understanding the social consequences of their behaviors. This can have far reaching consequences to people with ASD, such as not knowing when and why they make others upset^2–4^. Such difficulty hinders complete integration of people with ASD into family, classroom, and workplace^5–7^. However, it remains unknown what psychological mechanisms may underlie the impairments in understanding the social consequences of one’s own behaviors.

To appropriately respond emotionally and behaviorally to the social and interpersonal consequences caused by one’s behaviors, it is commonly proposed that one needs to have two intact systems: an affect recognition system that registers and infers the social partner’s negative affective states, and a situation understanding system that detects one’s responsibility in causing unpleasant outcomes^8^. Numerous studies with neurotypical participants have consistently demonstrated that these two cognitive processes are crucial for appropriate self-conscious emotions (e.g., guilt) and socially adaptive behaviors in this context^9,10^. In this model, the difficulty that people with ASD exhibit in this context could arise from impairments in either or both systems.

However, whether these two systems contribute to the altered emotional and behavioral responses of people with ASD in social interactions remains unclear. Although existing work has attempted to address whether and how the affect recognition system is impaired in people with ASD, evidence is not conclusive. Some evidence supports the notion that the affect recognition system functions as well in people with ASD as in neurotypical individuals^8^. Moreover, people with ASD also exhibit typical neural responses to emotional facial expressions, either isolated or embedded in a context, as measured with fMRI^11–14^. On the other hand, several impaired components of social communicative functioning have been reported, notably including impaired face processing and emotion recognition^1,15–21^.

It is similarly unclear whether people with ASD have impairments in situation understanding. Social interaction can be characterized by many situational factors, such as the relationship among interactive parties and social norms. A key function of the situation understanding system is to infer agency and responsibility. The ability to detect and attribute agency to oneself and to others is foundational to many social cognitive processes, such as assigning responsibility, inferring hidden mental states, and moral evaluations^22–24^. Research has shown that individuals with ASD do not exhibit impairments in the perception of agency in these non-social tasks^25,26^. Thus, neither the affective recognition system nor the situation understanding system may explain the atypical emotional and behavioral responses of people with ASD in social interactions. Here, we propose a novel, third system: social judgment system, which infers a social partner’s stable characteristics such as their social traits^27^. Prior research with neurotypical participants have demonstrated that judgments of others’ traits shape consequential decision-making, ranging from mating, voting, to courtroom sentencing^28–31^. Furthermore, people with ASD make different trait judgments from faces than neurotypicals, and that these differences become greater for more naturalistic, complex stimuli^1,27,32–34^.

In this study, we aimed to provide direct evidence for the link between the social judgment system and the atypical emotional and behavioral responses of people with ASD in social interactions. In particular, we examined whether people with ASD have a differential association between their social trait judgments of others from faces and their guilt experiences in interpersonal transgression contexts compared to neurotypical individuals. To characterize how people make social trait judgments from faces, we used naturalistic face stimuli of celebrities of diverse races, face angles, gaze directions, and facial expressions taken in naturalistic contexts (e.g., non-posing photos captured in the street or events)^35^ and a set of eight traits that summarize the comprehensive dimensions of trait judgments from faces^36^. To measure recognition of responsibility, guilt experience, and guilt-induced behavioral tendencies following interpersonal transgression, we adopted an interpersonal interaction task where we successfully manipulated participants’ responsibility in causing unpleasant outcomes to a social partner^37–40^. With these two tasks, we tested whether perception of social traits was associated with socio-emotional experience in interpersonal transgression context, and how such association was altered in people with ASD compared to neurotypical participants.

## Methods

### Participants

We recruited 230 participants from the Prolific platform. We only included participants who had English fluency, normal or corrected-to-normal vision, an education level above high school, and a Prolific approval rate greater than 95%. Fifty participants were excluded, leading to a sample of 180 participants for further analysis. Among the excluded participants, seven did not complete the interpersonal transgression task (see below), thirty-seven did not pass the comprehension check of the interpersonal transgression task, five missed at least an entire condition of the interpersonal transgression task, one's ASD diagnosis information is missing. Among the included participants, 44 participants had a self-reported diagnosis of ASD and 136 neurotypical participants reported no diagnosis of ASD and served as controls. Self-identification of ASD was probed by the following question in Prolific: “Have you received a formal clinical diagnosis of autism spectrum disorder, made by a psychiatrist, psychologist, or other qualified medical specialist? This includes Asperger’s syndrome, Autism Disorder, High Functioning Autism or Pervasive Developmental Disorder.” And we only included participants whose response was “Yes—as a child” or “Yes—as an adult” in the ASD group (not including any participants whose response was “I am in the process of receiving a diagnosis”, “No—but I identify as being on the autism spectrum”, “No”, or “Don’t know/rather not say”). Distinguishing the behavioral patterns of the two subgroups of self-identified ASD participants is beyond the scope of the present study. To make sure the two subgroups of self-identified ASD participants do not differ from each other on autistic traits, and that they both reported higher autistic traits than the neurotypical group, we conducted a one-way ANOVA on the AQ/SRS scores of the two subgroups of self-identified ASD participants, and neurotypical participants. There were significant differences between the three groups on both measures (AQ: *F*(2, 131) = 9.65, *p* < 0.001, η^2^ = 0.13; SRS: *F*(2, 131) = 5.90, *p* = 0.004, η^2^ = 0.08). Post-hoc Tukey tests revealed that the neurotypical group had significantly lower autistic scores than the adulthood diagnosed group (AQ: *p* = 0.001; SRS: *p* = 0.006) and the childhood diagnosed group (AQ: *p* = 0.020; SRS: *p* = 0.239). Importantly, the two subgroups of ASD participants did not differ in autistic traits (AQ: *p* = 0.861; SRS: *p* = 0.978). In the subsequent analysis, we collapse these two subgroups of self-identified ASD participants to obtain better statistical power.

Due to technical difficulties, only 33 ASD (18 female; mean age ± SD: 30.56 ± 8.95 years) and 124 neurotypical (47 female; 26.63 ± 7.54 years years) participants provided demographic information. These subsets of participants also completed a social trait judgment task (see below). To measure their ASD severity, we acquired Autism Spectrum Quotient (AQ) and Social Responsiveness Scale-2 Adult Self Report (SRS) from the participants (17 participants with ASD and 118 neurotypicals completed the questionnaires) and we confirmed that ASD participants had a significantly higher AQ (ASD: 28.18 ± 7.37 [mean ± SD], neurotypical: 20.53 ± 7.18; *t*(133) = 4.09, *p* < 0.001) and SRS (ASD: 86.76 ± 19.33, neurotypical: 64.84 ± 25.53; *t*(134) = 3.40,* p* < 0.001) than neurotypicals. Lastly, based on our screening criterion, neurotypicals had no mental health conditions. All participants provided written informed consent using procedures approved by the Institutional Review Boards of West Virginia University (Protocol #2012188080) and University of California Santa Barbara (Protocol # 2-22-0520). All methods adopted in this paper were performed in accordance with these protocols.

### Social trait judgment task

We used photos of celebrities from the CelebA dataset^35^, which has been described in detail elsewhere^32^. Participants provided judgments of social traits on a 7-point Likert scale. The social traits include *warm, critical, competent, practical, feminine, strong, youthful,* and *charismatic* as in previous studies^32,36^. For more details, please see Supplemental Methods.

### Interpersonal transgression task

**Figure 1:**
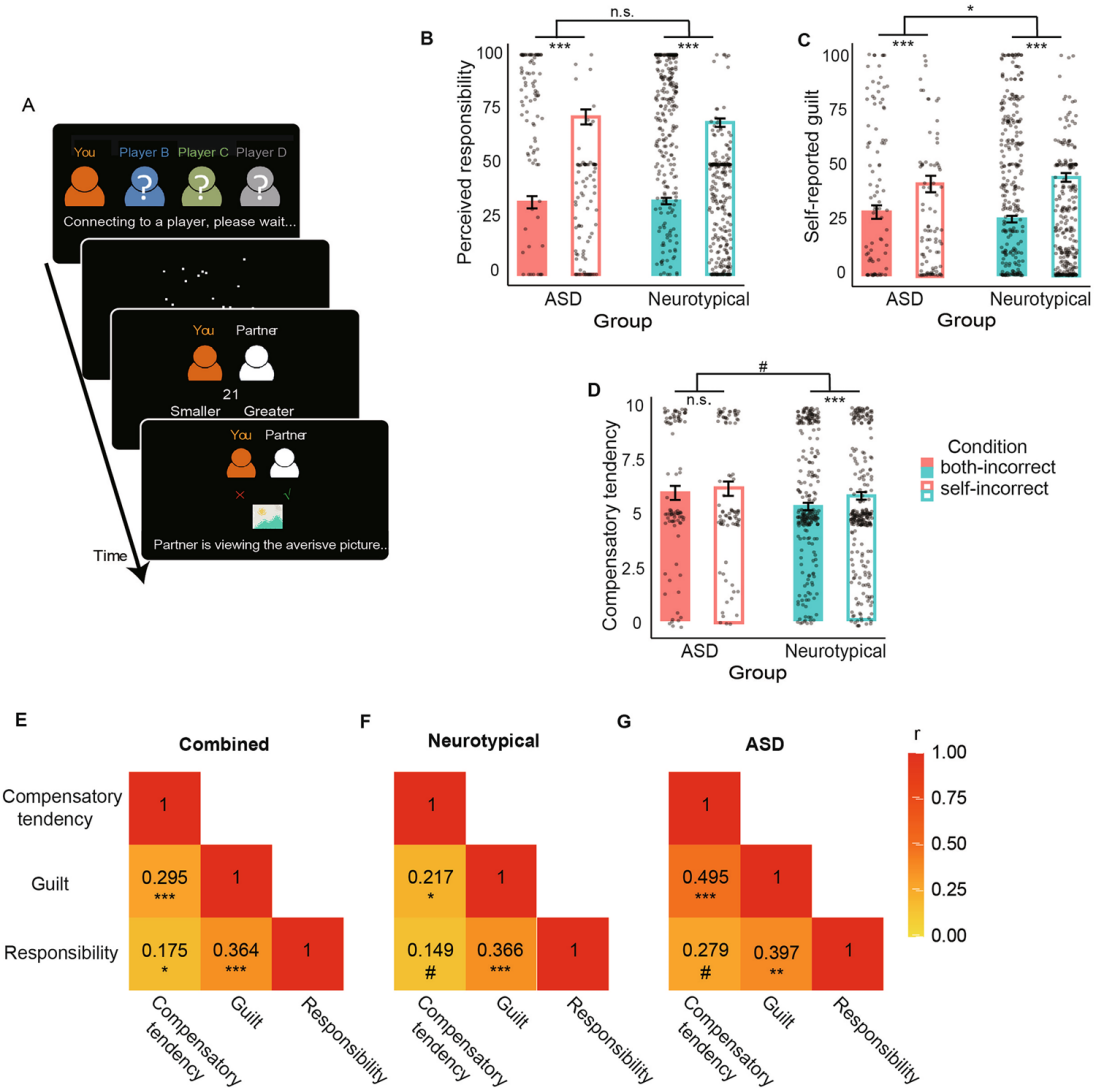
Procedure of the interpersonal transgression task and guilt-related measures. (**A**) Procedure of the guilt induction task. At the beginning of each trial, the participant was randomly paired with a player (i.e., Partner). The participant and the Partner then completed a dot estimation task, where they estimated the number of dots on the screen and compared it with a reference number (21 in this case). The participant’s and the Partner’s performance was then presented. Failure of either of them would lead to an unpleasant outcome for the Partner—viewing an aversive picture for 10 s. On different trials, participants were asked to report their perceived responsibility, feelings of guilt, and willingness to share the Partner’s unpleasant outcome as a hypothetical measure of compensation. (**B**) Perceived responsibility, (**C**) self-reported guilt, and (**D**) compensation tendency as a function of experimental conditions and participant groups. Significance of simple effects and group-by-condition interaction: ^#^*p* < 0.1, *p < 0.05, **p < 0.01 and ***p < 0.001. *n.s.* not significant. Error bars indicate s.e.m. (**E**–**G**) Correlations among the condition effects on perceived responsibility, self-reported guilt, and compensation tendency. ^#^p < 0.1, *p < 0.05, **p < 0.01 and ***p < 0.001.

In this online task, participants were paired with another hypothetical participant (hereafter, “partner”). On each trial (Fig. 1A), the participant and the partner saw an array of dots (about 20) displayed on the screen for a short interval (1.5 s). The participant and, ostensibly, the partner indicated whether the number of the dots was larger or smaller than a reference number (e.g., 20). Afterward, their performance was displayed on the screen. If one or both of them responded incorrectly, the partner had to watch an aversive image (i.e., unpleasant outcome), which was selected from the International Affective Picture System (IAPS)^41^. By manipulating the participant’s and the partner’s performance (i.e., correct/incorrect feedback), we were able to manipulate the participant’s responsibility in causing unpleasant outcomes to the partner. To make sure that the number of trials was balanced across conditions (i.e., both-correct, partner-incorrect, self-incorrect, both-incorrect), unbeknownst to the participants, the outcome feedback was predetermined. There were 6 trials for each of the four possible conditions. The order of the conditions was randomized across participants. Previous studies have demonstrated the validity of this task in inducing different levels of perceived guilt, negative self-conscious emotions, and compensatory behaviors^37–40,42^.

At the end of the trials where the partner had to watch the aversive picture, participants were prompted to answer one of following questions: (1) how guilty they were for the partner’s unpleasant outcome and (2) how long they would be willing to watch the aversive picture themselves, which could reduce the time the partner had to watch the aversive picture (i.e., a measure of compensation); (3) how much responsibility they believe they bear for their partners’ unpleasant outcome. Each question was randomly presented twice in each condition (excluding the both-correct condition), and participants indicated their responses on analog scales.

### Analytic plans

The aims of this study are to investigate: (1) the associations between the social trait judgment system and the social emotion system, and (2) whether such associations would differ in the ASD group relative to the neurotypical group, since ASD and neurotypical participants exhibited differential social trait judgment patterns (as reported elsewhere^32^, and here in Fig. S1; *Supplemental Results*). To achieve these aims, we plan to carry out the following statistical analysis.

First, since there has been very few empirical research on guilt experience and behavioral tendencies in ASD, let alone studies adopting an interactive task, we need to examine whether the manipulation of responsibility is modulated by participants’ group (self-identified ASD vs. neurotypical).

Second, we need to characterize individuals’ social trait judgment tendencies based on their social trait ratings across a large number of naturalistic face photos. We adopted two approaches to characterize social trait judgment tendencies. For the first approach, we averaged each participant’s ratings of a given social trait across all faces as the participant’s *central tendency* of their social trait judgment. For the second approach, we gauged the *sensitivity* of each participant’s social trait judgment. Specifically, we calculated a rank-based correlation (i.e., Spearman correlation) between each participant’s ratings of a given social trait across all faces and the average ratings of these same faces provided by a group of independent, neurotypical participants (N = 291). If we assume that the average ratings of the social traits of each face based on this independent group of participants reflect consensus signals of these traits conveyed by each face, then we could also interpret the correlation coefficients as reflecting how responsive or sensitive each participant was to these social traits. For guilt-related measure, we calculated the difference in self-reported guilt and compensatory tendency between the self-incorrect and both-incorrect conditions for each participant. The difference scores reflect the effects of responsibility on guilt experience and behavioral tendencies. We used these difference scores as the dependent variables in our linear regression models. Since we carried out statistical analysis separately for each of the eight social traits, we reported the original statistical results and the significance level after multiple comparisons correction. Here, we used the maximal permutation test to correct for multiple comparisons (see, e.g. Refs.^43–46^). This permutation-based method accounts for the potential interdependence across tests when correcting for multiple comparisons. In each permutation iteration, we randomly shuffled the guilt-related measures. We used this shuffled variable as the new dependent variable, which we fitted to the identical regression models as before. We then identified the maximum absolute t-value associated with the trait regressor across the eight trait models. This t-value serves as the maximal statistic based on the null hypothesis. This permutation procedure was iterated 1000 times, resulting in distributions of the largest possible null effect for the trait main effect and trait-by-group interaction on self-reported guilt and compensatory tendency, respectively. We then calculated the corrected *p*-value for each model by comparing the observed absolute *t*-value of regression coefficient with the correspondent null distribution.

As a further exploratory analysis, we delved deeper into the relationship between individual differences in perceiving a face as critical and the individual differences in guilt-related psychological processes, as well as how ASD and neurotypical groups differ in this regard. The rationale of focusing on judgments of criticalness is due to the interpersonal nature and social adaptive function of guilt. From an adaptationist’s perspective, guilt is an emotional signal that indicates potential social threat and scrutiny from the victim of one’s transgression. It is thus conceivable that a more formidable and critical victim has more potentials to incur more social consequence towards the transgressor, and thus elicit stronger guilt experience and behavioral motivations to make amend^47–49^. Here, we conducted an inter-subject representational similarity analysis (IS-RSA) as an alternative approach to examine the associations between the individual differences in criticalness judgment and guilt-related processes^50–52^. To this end, we first constructed representation dissimilarity matrices (RDMs) based on each individual differences measure (i.e., condition effects on self-reported guilt and compensatory tendency, mean criticalness rating). Specifically, each RDM consists of the absolute difference in an individual differences measure between all possible pairs of participants (within each group). We then ran linear regression models where the RDMs based on guilt-related measures were entered as the dependent variable and the RDM based on *critical* judgment was entered as the predictor. The main effect of group and the group-by-trait interaction were also included. A positive regression coefficient of the *critical* RDM would indicate that two participants who are similar in their *critical* judgment would also be similar in their guilt-related measures. A significant group-by-trait interaction would suggest the above association differ between the two groups. This analysis would reveal a different aspect of individual differences compared to the standard regression analysis described above (for details, see *Inter-subject representational similarity analysis (IS-RSA)* in the *Supplementary Methods*).

## Results

### Self-reported guilt experience and compensatory tendency as a function of responsibility

We first examined the two groups’ performance in the interpersonal transgression task. We focused on comparing the self-incorrect condition and the both-incorrect condition because the participants’ performance feedback was the same in these two conditions. Therefore, participants’ perception of their own performance (i.e., correct versus incorrect) should not have any impact on the comparison. The only difference between the two conditions was the participants’ responsibility in causing the unpleasant outcome to the partner—while in the self-incorrect condition the participant was fully responsible, in the both-incorrect condition the partner and the participant shared the responsibility, thereby diluting it^39^.

As a manipulation check, we examined if the participants perceived higher responsibility in the self-incorrect condition than the both-incorrect condition (Fig. 1B). This was exactly what we found—participants reported higher responsibility in the self-incorrect condition than in the both-incorrect condition (linear mixed effect model; *B* = 36.21 ± 1.45, 95% CI [33.37, 39.05]; *b* = 1.08, 95% CI [0.99, 1.16]; *t* = 25.00, *p* < 0.001). There was no significant difference in perceived responsibility between groups (*B* = − 1.17 ± 3.93, 95% CI [− 8.87, 6.54]; *b* = − 0.03, 95% CI [− 0.26, 0.20];* t* = − 0.30, *p* = 0.767).

We next examined the condition effect on self-reported guilt and compensatory tendency. We found that overall, the participants reported experiencing more guilt in the self-incorrect condition than in the both-incorrect condition (*B* = 17.51 ± 1.35, 95% CI [14.86, 20.17]; *b* = 0.57, 95% CI [0.48, 0.65]; *t* = 12.95, *p* < 0.001). Overall self-reported guilt did not differ significantly between groups (*B* = 0.10 ± 4.36, 95% CI [−8.44, 8.65]; *b* = 0.003, 95% CI [− 0.27, 0.28]; *t* = 0.02, *p* = 0.981). Compensatory tendencies showed a similar pattern, which was significantly higher in the self-incorrect condition than the both-incorrect condition (*B* = 0.41 ± 0.09, 95% CI [0.24, 0.60]; *b* = 0.14, 95% CI [− 0.49, 0.15]; *t* = 4.63, *p* < 0.001). Similarly, there was no significant difference in overall compensatory tendency between the two groups (*B* = − 0.53 ± 0.50, 95% CI [− 1.52, 0.46]; *b* = − 0.17, 95% CI = [− 0.49, 0.15]; *t* = − 1.05, *p* = 0.295).

We next explored whether the effect of condition (i.e., responsibility manipulation) differed between the ASD group and the neurotypical group. To this end, we ran a separate set of linear mixed effect models where we additionally included the group-by-condition interaction term. For self-reported guilt, the experimental condition had stronger effects in the neurotypical group than in the ASD group, as evident by the significant group-by-condition interaction for self-reported guilt (Fig. 1C; *B* = 7.32 ± 3.13, 95% CI [1.18, 13.46]; *b* = 0.24, 95% CI [0.04, 0.44]; *t* = 2.34, *p* = 0.020) and, to a lesser degree, the tendency towards compensating the partner (i.e., compensatory tendency; Fig. 1D; *B* = 0.36 ± 0.21, 95% CI [− 0.05, 0.77]; *b* = 0.12, 95% CI [− 0.02, 0.25]; *t* = 1.74, *p* = 0.083), but not for self-reported responsibility (*B* = − 2.67 ± 3.36, 95% CI [− 9.25, 3.91]; *b* =  − 0.08, 95% CI [− 0.28, 0.12]; *t* =  − 0.80, *p* = 0.427). Specifically, for self-reported guilt, condition had a larger effect in the neurotypical group (*B* = 19.30 ± 1.61, 95% CI = [16.14, 22.45]; *b* = 0.64, 95% CI [0.53, 0.74]; *t* = 12.01, *p* < 0.001) than in the ASD group (*B* = 12.34 ± 2.42, 95% CI [7.58, 17.10]; *b* = 0.38, 95% CI [0.23, 0.52]; *t* = 5.10, *p* < 0.001). For compensatory tendency, the neurotypical group also showed a stronger condition effect (*B* = 0.49 ± 0.11, 95% CI [0.28, 0.70]; *b* = 0.16, 95% CI [0.09, 0.23]; *t* = 4.59, *p* < 0.001) than the ASD group (*B* = 0.19 ± 0.16, 95% CI [− 0.13, 0.50]; *b* = 0.06, 95% CI [− 0.04, 0.15]; *t* = 1.17, *p* = 0.246).

These results suggest that responsibility in causing unpleasant outcomes to a social partner had less impact on ASD participants’ guilt experience and behavioral responses compared with neurotypical participants.

### Relationships among guilt-related psychosocial processes

It has been theorized that perceived responsibility in causing harm to another person is an important antecedent of guilt^9,39,53,54^. Moreover, feeling of guilt has also been demonstrated as a motivational force behind compensatory and reparatory behaviors^9,39,40,55,56^. We therefore explored the relationships among the three guilt-related processes, and whether these relationships differ between the neurotypical and the ASD groups. We first calculated condition effects on perceived responsibility, self-reported guilt, and compensatory tendency as the difference between the self-incorrect and both-incorrect conditions. Then we obtained the correlation coefficients between all three pairs of condition effects. We did this for all participants (Fig. 1E), and separately for the neurotypical participants (Fig. 1F), and for the ASD participants (Fig. 1G). As the figure illustrates, most of these correlations were statistically significant, and the correlations did not differ significantly between the neurotypical participants and the ASD participants.

### Guilt-related processes and autistic tendencies

In the above analyses, we treated group (ASD vs. neurotypical) as a binary variable. However, autistic tendency is a continuous distribution in both clinical and subclinical populations. In an exploratory analysis, we asked if guilt-related processes are correlated with continuous autistic tendencies. We collapsed the two groups and examined the correlations between the continuous autistic tendencies (AQ and SRS scores; Fig. S2A and B) and the difference in self-reported guilt and compensation tendency between self-incorrect condition and both-incorrect condition. Neither AQ nor SRS showed significant correlation with difference in self-reported guilt (AQ: *r* = − 0.02, *p* = 0.781; SRS: *r* = 0.02, *p* = 0.814; Fig. S2C and D), or difference in compensation tendency (AQ: *r* = 0.08, *p* = 0.280; SRS: *r* = − 0.01, *p* = 0.879; Fig. S2E and F). This could be because participants with high autistic tendencies were underrepresented in our sample (Fig. S2A and B). Future research with larger samples and larger diversity in autistic tendencies is needed to ascertain the relationship between continuous autistic tendencies and guilt-related processes.

### The associations between guilt-related measures and social trait judgment tendencies

We next investigated whether individual differences in guilt-related psychological processes (e.g., guilt experience and compensatory tendency) were associated with individual differences in social trait judgments. As described in “Methods”, we adopted two approaches to characterize participants’ social trait judgment tendencies. Below we reported regression results based on each of these approaches.

#### Central tendency in social trait judgments

As detailed in the *Analytic Plan* section in “Methods”, we first ran linear models with condition effects (i.e., self-incorrect vs. both-incorrect) on self-reported guilt and compensatory tendency as the dependent variable, and mean social trait judgments (one trait in each separate model) and participants’ group as the predictors. We found that judgments of *critical* was positively associated with difference in guilt (*B* = 6.88 ± 2.64, 95% CI [1.67, 12.09]; *b* = 0.21, 95% CI [0.05, 0.37]; *t* = 2.61, *p* = 0.010). To further explore the trait-by-group interaction effect on guilt processing, we included the interaction term in a new regression model, which revealed a significant trait-by-group interaction for judgments of *charismatic* (*B* = − 14.54 ± 6.53, 95% CI [− 27.44, − 1.64]; *b* = − 0.42, 95% CI [− 0.78, -0.05]; *t* = − 2.23, *p* = 0.027; Fig. S3A), such that the tendency to perceive a face as *charismatic* in the neurotypical group is positively associated with self-reported guilt difference (*B* = 6.10 ± 3.29, 95% CI [− 0.42, 12.62]; *b* = 0.17, 95% CI [− 0.01, 0.35]; *t* = 1.85, *p* = 0.066), but this association was in the opposite direction in the ASD group (although not statistically significant; *B* =  − 8.44 ± 5.02, 95% CI [− 18.68, 1.80]; *b* =  − 0.29, 95% CI [− 0.64, 0.06]; *t* =  − 1.68, *p* = 0.103). A similar trait-by-group interaction pattern was observed for *practical* (*B* = − 13.47 ± 6.48, 95% CI = [− 26.26, − 0.67]; *b* =  − 0.38, 95% CI [− 0.73, − 0.02]; *t* =  − 2.08, *p* = 0.039; Fig. S3B).

For difference in compensatory tendency, we found a weak positive association with judgments of *competent* (*B* = 0.36 ± 0.19, 95% CI [− 5.11, 5.82]; *b* = 0.01, 95% CI [− 0.15, 0.17]; *t* = 1.92, *p* = 0.056), indicating that the inclination of perceiving others as competent is positively related to compensatory tendency.

#### Sensitivity in social trait judgments

The above analyses showed that the central tendencies in social trait judgments were associated with self-reported guilt and compensatory tendency. Next, we investigated whether individual differences in the sensitivity in social trait judgments were associated with guilt-related psychological processes. As we described in the *Analytic Plan* section, sensitivity for each social trait was calculated as the rank-based correlations between each participant’s trait ratings across all the faces and an independent group of participants’ average ratings across the same faces. ASD and neurotypical groups did not differ in the sensitivity in social traits judgments except for the judgment of *competent* (Mann–Whitney* U* test, *W* = 1263, *p* = 0.014). We next examined the associations between sensitivity in social trait judgments and individual differences in condition effects on self-reported guilt and compensatory tendencies.

For self-reported guilt, we found a significant trait-by-group interaction for the sensitivity in *critical* judgment (*B* =  − 69.27 ± 28.77, 95% CI [− 126.18, − 12.37]; *b* =  − 0.45, 95% CI [− 0.82, − 0.08]; *t* =  − 2.41, *p* = 0.017; Fig. S3C). Specifically, for the neurotypical group, higher sensitivity in the judgment of *critical* was associated with greater self-reported guilt difference (*B* = 23.26 ± 9.97, 95% CI [3.53, 42.99]; *b* = 0.21, 95% CI [0.03, 0.39]; *t* = 2.33, *p* = 0.021). In contrast, for the ASD group, the association was in the opposite direction but did not reach statistical significance (*B* =  − 46.37 ± 23.11, 95% CI [− 95.92, 3.19]; *b* =  − 0.47, 95% CI [− 0.98, 0.03]; *t* =  − 2.01, *p* = 0.065).

For compensatory tendency, we found a significant main effect of the sensitivity in the *warm* judgment (*B* = 1.62 ± 0.68, 95% CI [0.28, 2.96]; *b* = 0.20, 95% CI [0.03, 0.36]; *t* = 2.39, *p* = 0.019), indicating that individuals who are more sensitive to warmth signals in others’ faces are also more likely to compensate when causing unpleasant outcomes to others, and this effect did not differ between the neurotypical and ASD group (trait-by-group interaction: *B* = − 32.36 ± 40.52, 95% CI [− 112.49, 47.78]; *b* =  − 0.21, 95% CI [− 0.72, 0.30]; *t* =  − 0.80, *p* = 0.426). Interestingly, we found a significant trait-by-group interaction for the sensitivity in *competent* judgment (*B* = 7.98 ± 3.11, 95% CI [1.83, 14.12]; *b* = 0.94, 95% CI [0.22, 1.67]; *t* = 2.57, *p* = 0.011; Fig. S3D). Specifically, the sensitivity in *competent* judgment was positively associated with compensatory tendency in the ASD group (*B* = 7.38 ± 3.14, 95% CI [0.60, 14.16]; *b* = 0.55, 95% CI = [0.04, 1.05]; *t* = 2.35, *p* = 0.035), but not in the neurotypical group (*B* =  − 0.59 ± 0.75, 95% CI [− 2.07, 0.88]; *b* =  − 0.07, 95% CI [− 0.25, 0.11]; *t* =  − 0.80, *p* = 0.428).

The maximal permutation tests used to estimate significance level after correction for multiple comparisons indicated that the main effect of the association between the central tendency of criticalness judgment and guilt difference was trending (*p* = 0.060). All the other effects in the central tendency and sensitivity regression models did not survive the correction. Therefore, the conclusions based on the above analyses should be taken with a grain of salt. We additionally carried out robustness check using an alternative analysis of individual differences, namely, inter-subject representational similarity analysis (IS-RSA).

### Inter-subject representational similarity analysis (IS-RSA)

**Figure 2:**
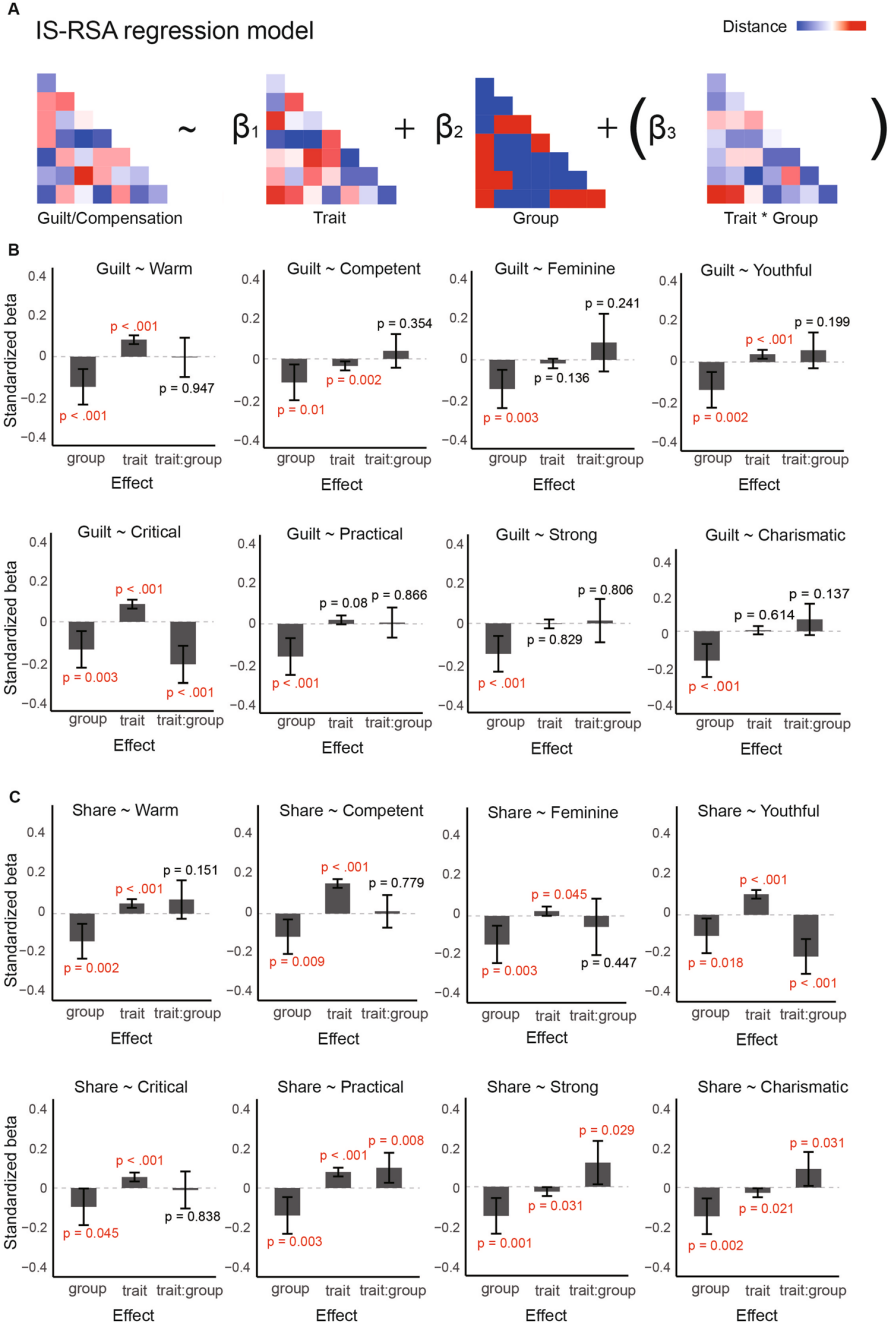
Inter-subject representational similarity analysis (IS-RSA). (**A**) The conceptual scheme of regressions based on representational dissimilarity matrix (RDM). To explore the interaction between individual differences in social trait judgment and group, we ran separate models that included the trait-by-group interaction term. (**B**) Results of regressions predicting the RDM of self-reported guilt difference. (**C**) Results of regressions predicting the RDM of compensatory tendency (i.e., “Share”) difference.

To further explore the relationship between individual differences in perceiving a face as critical and the individual differences in guilt-related psychological processes, as well as how ASD and neurotypical groups differ in this regard, we conducted an inter-subject representational similarity analysis (IS-RSA) (Fig. 2A). We carried out this analysis in two manners. For the association between central tendency of criticalness judgment and self-reported guilt difference, we used IS-RSA as a confirmatory analysis, since the above analysis indicated a hint of their association. For completeness, we used IS-RSA to replicate all the other analyses reported in the above section. These analyses were exploratory, therefore we carried out maximal permutation tests reported above to estimate the significance level after multiple comparisons correction.

#### Confirmatory analysis

We found an overall significant positive association between *critical* RDM and self-reported guilt difference RDM (*B* = 2.79 ± 0.36, 95% CI [2.08, 3.49]; *b* = 0.09, 95% CI of *b* = [0.07, 0.11]; *t* = 7.72, *p* < 0.001). This indicates that similarity in *critical* judgments is positively associated with similarity in self-reported guilt difference. To further explore the association between *critical* judgment and guilt in different groups, we included a group-by-trait interaction in the regression model, which was significant (*B* =  − 6.71 ± 1.49, 95% CI [− 9.63, -3.79]; *b* =  − 0.21, 95% CI of *b* = [− 0.30, − 0.12]; *t* =  − 4.51, *p* < 0.001; Fig. 2B). While similarity in *critical* RDM was positively associated with self-reported guilt RDM in the neurotypical group (*B* = 3.21 ± 0.38, 95% CI [2.47, 3.94]; *b* = 0.10, 95% CI of *b* = [0.08, 0.12]; *t* = 8.55, *p* < 0.001), the opposite relationship was observed in the ASD group (*B* =  − 3.50 ± 1.26, 95% CI [− 5.98, − 1.03]; *b* =  − 0.12, 95% CI of *b* = [− 0.21, − 0.04]; *t* =  − 2.78, *p* = 0.006). To further validate these effects, we conducted a permutation test comparing the actual coefficients to a null distribution generated by 1000 iterations. This test confirmed the significance of the group-by-trait interaction (observed *B* =  − 6.71, 95% CI of null coefficient = [− 6.11, 6.36], *p* = 0.006) and, to a lesser degree, the main effect (observed *B* = 2.79, 95% CI of null coefficient = [− 3.02, 3.03], *p* = 0.090).

#### Exploratory analysis

For difference in compensatory tendency (Fig. 2C), we also found a significant main effect of the *critical* RDM (*B* = 0.15 ± 0.03, 95% CI [0.09, 0.21]; *b* = 0.05, 95% CI of *b* = [0.03, 0.08]; *t* = 4.81, *p* < 0.001), but not the group-by-trait interaction (*B* =  − 0.03 ± 0.12, 95% CI [− 0.28,0.22]; *b* < 0.01, 95% CI of *b* = [− 0.10, 0.08]; *t* =  − 0.21, *p* = 0.838). Figure 2B and C showed the associations between all the social trait judgments and guilt-related processes (i.e., self-reported guilt differences and compensation differences). However, the maximal permutation tests indicated that none of the exploratory results survived multiple comparisons correction.

## Discussion

In this study, we showed that social trait judgments from faces were associated with socio-emotional experience in social interactions: they were linked to guilt following interpersonal transgression, but such effect was weakened in individuals with ASD. The present study has advanced our understanding of this question in at least two ways.

From a theoretical perspective, our study provides novel understanding regarding the link between the social judgment system and social emotion in ASD. A social partner’s face is a rich source of social-affective information^57^. Past research in social psychology and affective sciences has suggested that how a transgressor perceives the victim’s face is crucial to the transgressor’s emotional experience following the transgression^38,58^. Here, we found that, consistent across several analytic approaches and the two groups, one’s tendency to perceive others’ face as being critical is positively associated with their propensity to feel guilt for harming others. This result is in line with the adaptationist view of guilt, which posits guilt as an emotional signal indicating that one has disappointed a valuable social partner^47,59^. Value here refers to the social partner’s ability in influencing the social and physical well-being of the transgressor, and previous research has demonstrated that a transgressor would feel more guilt when harming someone who can determine their (i.e., the transgressor’s) future payoff^60^. A more critical victim is more likely to hold an unforgiving and vengeful attitude towards the transgressor compared with a warm victim^61^. It is thus conceivable that in our study, the participants who tended to perceive others’ faces as more critical exhibited stronger guilt signals. Interestingly, both the sensitivity and the IS-RSA shows that the association between criticalness judgment and self-reported guilt is different in ASD participants compared with neurotypical participants. This distinct pattern suggests that individuals with ASD may have altered link between perceiving social partners’ properties (e.g., criticalness vs. warmth) and social and affective responses in the interactions with the social partners^4,6,7^. We acknowledge the correlational nature of the association analysis. Future studies that directly manipulate social traits of the victim are needed to further delineate the causal role of social trait perception in guilt.

From a methodology perspective, our study is among the first to adopt a social interactive task to investigate social emotions in ASD. Most prior research in this field has adopted social observation and imagination task, such as passively viewing picture and/or videos, or reading hypothetical vignettes^62–64^. Imagining a social interaction from the third-person perspective has been shown to rely on distinct neurocognitive processes compared to engaging in a social interaction from the first-person perspective^65–69^. Recent research on the neural bases of social emotions have developed interpersonal interactive tasks that allow the participants to interact with real social partners^39,40,66,70–72^. These interactive tasks allow the researchers to elicit and measure social-affective processes and their behavioral tendencies in naturalistic social contexts and pinpoint the underlying neurocognitive processes. Here, we adapted this interpersonal interactive task to an online testing environment and provided a preliminary yet promising result about the link between social trait perception and social emotions. Moreover, we adopted two complementary approaches to characterize an individual’s social trait perception tendency. The central tendency captures one’s overall propensity in perceiving a social trait across different faces, while the sensitivity captures how responsive one is in perceiving a social trait from different faces. Our results reveal both consistent and distinct associations between trait perception and guilt-processes when using central tendency and sensitivity. Of note, in neurotypical participants at least, both the central tendency and sensitivity of criticalness judgment is positively associated with self-reported guilt, suggesting a crucial role of criticalness judgment in the appraisal of guilt. In contrast, the sensitivity, but not the central tendency of warmth judgment is positively associated with compensation behavior, indicating those who are more sensitive to warm faces are more willing to compensate the harm they inflict on others.

There are several limitations that future studies need to address. First, the participants in the ASD group were recruited from an online platform, and we could not independently verify their diagnosis status. However, studies have demonstrated the validity and utility of studying clinical samples from online crowdsourcing platforms^73^. Future work with clinically diagnosed sample is needed to replicate and confirm the current findings. Relatedly, our interpersonal transgression task had to be adapted to fit the online format, which inevitably compromised the interactive nature of the task. Future research is needed to replicate and extend this finding in a more life-like environment (e.g., a laboratory setting with real-time interactions with human partners).

In this study, we used celebrity faces as stimuli, and it is important to acknowledge that these faces may differ from those typically encountered in terms of familiarity, attractiveness, and various other factors. For example, prior knowledge of familiar faces can influence face perception, particularly in the context of social trait judgment^74–76^, and familiar faces may engage distinct neural processes when compared to unfamiliar faces^77^. Furthermore, the level of familiarity can vary among stimuli and across participants. While the celebrity faces employed in our study were familiar, it is pertinent to note that the self-reported familiarity did not differ between ASD and neurotypical groups, and the celebrities may not have held personal relevance for the participants. The familiarity associated with celebrity faces might have been weaker compared to that of personally relevant and familiar faces, such as photographs featuring the participants themselves, their families, or the experimenters. Despite this, it is important to emphasize that we could replicate our results in social trait judgments using celebrity faces that participants were not familiar with as well as more controlled face stimuli featuring unfamiliar individuals with neutral expressions, direct gaze, and uniform backgrounds^32^.

In sum, by combining a comprehensive social trait judgment stimulus set with an interpersonal transgression task, we show that compared with neurotypical individuals, individuals with ASD exhibited weaker effect of responsibility on guilt and compensatory behaviors. People with ASD also show different associations between social trait judgment from faces and guilt, which provides a novel account for their altered guilt responses.

### Supplementary Information

Supplementary Information.

## Data Availability

The data and codes needed to reproduce the analyses reported in this paper can be found on this OSF account: https://osf.io/3xsn9/.
