# Supplementary material for: Differences in the link between social trait judgment and socio-emotional experience in neurotypical and autistic individuals
Source: Sci Rep. 2024 Mar 5;14:5400. doi: 10.1038/s41598-024-56005-5 (PMC10915137; doi:10.1038/s41598-024-56005-5)
Supplement: Supplementary file 1 — Supplementary Information. [file 41598_2024_56005_MOESM1_ESM.docx]

*Supplementary Information for*

**Differences in the link between social trait judgment and socio-emotional experience in neurotypical and autistic individuals**

**Supplementary Methods**

*Social trait judgment task*

We used photos of celebrities from the CelebA dataset^1^. We selected a total of 500 photos of 50 identities, each identity with 10 photos. The 50 identities included both sexes (33 male) and multiple races (40 identities were Caucasian, 9 identities were African American, and 1 identity was biracial). All photos were taken in naturalistic contexts, with faces displaying various expressions, head poses, and gaze directions, surrounded by various backgrounds and lighting. Participants provided judgments of social traits on a 7-point Likert scale. The social traits include *warm, critical, competent, practical, feminine, strong, youthful,* and *charismatic*. We selected these eight social traits to represent the comprehensive dimensions of trait judgments from faces found in prior research^2^. Participants also indicated whether they recognized the identity of the face (i.e., whether they were familiar with each face identity). We did not find a significant correlation between the percentage of familiar identities and AQ (ASD: *r*(15) = −0.18, *p* = 0.48; neurotypicals: *r*(116) = −0.07, *p* = 0.47) nor SRS (ASD: *r*(15) = −0.18, *p* = 0.50; neurotypicals: *r*(116) = 0.03, *p* = 0.78), suggesting the two groups were similarly (un)familiar with the faces. The 500 images were divided into 10 modules, with each module containing one face image for each of the 50 identities. Participants rated all the faces on all the eight social traits (rated in blocks) in each module.

We applied the following three exclusion criteria: (1) Trial-wise exclusion: we excluded trials with reaction times shorter than 100 ms or longer than 5000 ms. (2) Block/trait-wise exclusion: we excluded the entire block per module if more than 30% of the trials were excluded from the block per (1) above, or if there were fewer than 3 different rating values in the block (this suggests that the participant may not have used the rating scale properly). (3) Module-wise exclusion: we excluded a module if more than 3 blocks were excluded from the module per (2) above.

*Inter-subject representational similarity analysis (IS-RSA)*

To explore the association between individual differences in social trait judgments and guilt processing, we conducted an inter-subject representational similarity analysis (IS-RSA). In the IS-RSA, we compared the dissimilarity between all pairs of participants’ trait judgments and self-reported guilt as well as compensatory tendency, representing by lower triangular dissimilarity matrices (DMs) that quantify the dissimilarity between all pairs of participants.

First, we built a DM for trait judgments by calculating the absolute difference in mean rating scores for each pair of participants across the 500 images. We then built a corresponding DM for guilt difference and compensatory tendency difference (i.e., difference between self-incorrect and both-incorrect condition) based on the absolute differences in self-reported guilt for each pair of participants. In a DM, larger values represent greater dissimilarity between pairs, with the smallest possible value being the similarity of a condition unto itself (a dissimilarity of 0).

To compare the pattern similarity between each social trait and guilt, we converted the DMs into column vectors. We then conducted a linear regression with guilt dissimilarity as the dependent variable and trait dissimilarity, group as independent variables. To further explore the interaction between trait and group, we built models that included the trait-by-group interaction. We further used a permutation test with 1000 iterations to build a null distribution to statistically compare the observed regression coefficients with those generated from surrogate data. In each iteration, we shuffled the participant labels, recalculated the guilt DM, and refit the regression model based on this surrogate guilt DM. We then compared the actual coefficients to the null distribution.

**Supplementary Results**

*Social trait judgments in ASD and NT samples*

We confirmed that in our sample, participants with autism spectrum disorder (ASD) made different social trait judgments from faces than neurotypical (NT) participants, consistent with prior research^3^. Half of the ASD participants who completed our social trait judgment task were overlapping with those in the prior study^3^, and that our neurotypical participants were a subset of participants in that study. We showed that participants with ASD demonstrated reduced specificity in social trait judgments (**Fig. S1**) as shown in the previous study^3^. Specifically, we arranged the different face identities according to how they were rated by neurotypical participants, based on their average ratings (shown in **Fig. S1A**). We found that individuals with ASD gave less varied ratings across the different face identities compared to neurotypical participants, particularly for judgments of *competent*, *practical*, *feminine*, and *youthful* (as illustrated in **Fig. S1A, D**). This resulted in higher ratings for faces that neurotypicals rated low, and lower ratings for faces that neurotypicals rated high (for example, competence and practicality in **Fig. S1A**). To quantify this finding, we compared the ratings given by each group separately for the 10 face identities that neurotypicals rated highest (**Fig. S1B**) and the 10 face identities that neurotypicals rated lowest (**Fig. S1C**). We found that compared to neurotypicals, participants with ASD provided significantly lower ratings for the top 10 identities when judging *critical* (**Fig. S1B**; *t*(240) = 2.15, *p* = 0.032), *competent* (*t*(234) = 2.87, *p* = 0.0045), *feminine* (*t*(222) = 4.46, *p* = 1.32×10^−5^), and *youthful* (*t*(246) = 2.69, *p* = 0.076); and they provided significantly higher ratings for the bottom 10 identities for *competent* (**Fig. S1C**; *t*(234) = 3.03, *p* = 0.0027), *practical* (*t*(240) = 2.66, *p* = 0.0084), *feminine* (*t*(222) = 4.83, *p* = 2.60×10^−6^), and *strong* (*t*(245) = 2.00, *p* = 0.046). Therefore, the difference between the top 10 and bottom 10 identities was significantly smaller in participants with ASD compared to neurotypicals in these traits (**Fig. S1D**): *competent* (*t*(234) = 5.99, *p* = 7.83×10^−9^), *practical* (*t*(240) = 3.09, *p* = 0.0022), *feminine* (*t*(222) = 5.82, *p* = 2.05×10^−8^), and *youthful* (*t*(246) = 2.01, *p* = 0.046). The findings suggest that people with ASD have a reduced capacity to judge social traits from faces. This is consistent with prior studies showing that people with ASD have a reduced specificity in recognizing emotions from faces^4^ as well as more irregular and unpredictable eye movements when looking at faces^5–7^.

*Robustness check for the association between guilt-related measures and social trait judgment tendencies*

To buttress the regression analysis reported in the main text, we adopted a different format of linear model. Specifically, we included the self-reported guilt and compensatory tendency in the self-incorrect condition as the dependent variable. For predictors, as in the regression reported in the main text, we first included each participant’s mean social trait judgments (one in each separate model), participants’ group. Additionally, we included the self-reported guilt and compensatory tendency in the both-incorrect condition as a control variable. For exploration purpose, we further built regression models included the trait-by-group interaction term.

For the models of self-reported guilt, confirming the findings reported in the main text, we found a significant main effect of *critical* (*B* = 7.07±2.66, 95% CI = [1.80, 12.33]; *b* = 0.15, 95% CI = [0.04, 0.26]; *t* = 2.65, *p* = 0.009). We further included the trait-by-group interaction term and observed a significant trait-by-group interaction for *charismatic* (*B* = −13.39±6.45, 95% CI = [−26.13, −0.64]; *b* = −0.27, 95% CI = [−0.53, −0.01]; *t* = −2.08, *p* = 0.040), such that the tendency to perceive a face as charismatic in the neurotypical group was positively associated with reporting guilt when one was solely responsible for harming another (*B* = 6.89±3.34, 95% CI = [0.27, 13.51]; *b* = 0.14, 95% CI = [0.01, 0.27]; *t* = 2.06, *p* = 0.042), but this association was in the opposite direction in the ASD group (although not statistically significant; *B* = −6.65±4.99, 95% CI = [−16.83, 3.52]; *b* = −0.14, 95% CI = [−0.36, 0.07]; *t* = −1.33, *p* = 0.192). A similar trait-by-group interaction pattern was observed for *practical* (*B* = −13.86±6.50, 95% CI = [−26.71, −1.02]; *b* = −0.27, 95% CI = [−0.53, −0.02]; *t* = −2.13, *p* = 0.035). That is, the associations between self-reported guilt and these social trait judgments were weaker in participants with ASD compared to neurotypicals. For a neurotypical participant, the more they tend to judge a face as practical and charismatic, the more sensitive their guilt experience is to the responsibility in causing unpleasant outcomes to others. This association was absent in the ASD group.

For the models of compensatory tendency, confirming the findings reported in the main text, we found a significant main effect of *competent* (*B* = 0.40±0.18, 95% CI = [0.04, 0.76]; *b* = 0.08, 95% CI = [0.01, 0.16]; *t* = 2.17, *p* = 0.031).

Taken together, these results suggest that participants with ASD have altered association between the ability to infer social trait information from others and the ability to recognizing their agency in social interactions. Adopting different forms of the regression model did not alter our main findings.

*Replicate results on a balanced sample*

To address the issue of imbalanced group sizes, we matched participants with ASD and neurotypical controls using the 'MatchIt' package in R. This matched each ASD participant to a neurotypical participants based on sex and age, resulting in a new pseudo-sample (n = 66) with equal size and equivalent demographic features (sex: *χ^2^*(1) = 0, *p* = 1; age: *W* = 545, *p* = 1) between groups. Our major findings were largely replicated in the matched sample.

**The association between guilt-related measures and central tendency in social trait judgments.** We successfully replicated the main effect of *critical* (*B* = 8.07 ± 4.15, 95% CI = [-0.23, 16.38]; *b* = 0.24, 95% CI = [-0.01, 0.49]; *t* = 1.94, *p* = 0.057; marginally significant), the trait-by-group interaction of *charismatic* (*B* = -19.94 ± 8.89, 95% CI =[-37.72, -2.17]; *b* = -0.55, 95% CI =[-1.04, -0.06]; *t* = -2.24, *p* = 0.029) and *practical* (*B* = -19.56 ± 8.79, 95% CI =[-37.44, -2.27]; *b* = -0.56, 95% CI =[-1.06, -0.06]; *t* = -2.26, *p* = 0.028) on self-reported guilt difference.

**The association between guilt-related measures and sensitivity in social trait judgments.** We replicated the trait-by-group interaction effect of *critical* sensitivity on self-reported guilt (*B* = -66.74 ± 38.44, 95% CI = [-144.16, 10.68]; *b* = -0.53, 95% CI =[-1.15, 0.09]; *t* = -1.74, *p* = 0.089) and the interaction effect of *competent* sensitivity on compensatory tendencies (*B* = 8.23 ± 4.01, 95% CI = [0.14, 16.31]; *b* = 0.80, 95% CI =[0.01, 1.59]; *t* = 2.05, *p* = 0.046).

These results suggest that imbalanced sample sizes did not substantially influence our major findings.

**Supplementary Figure**


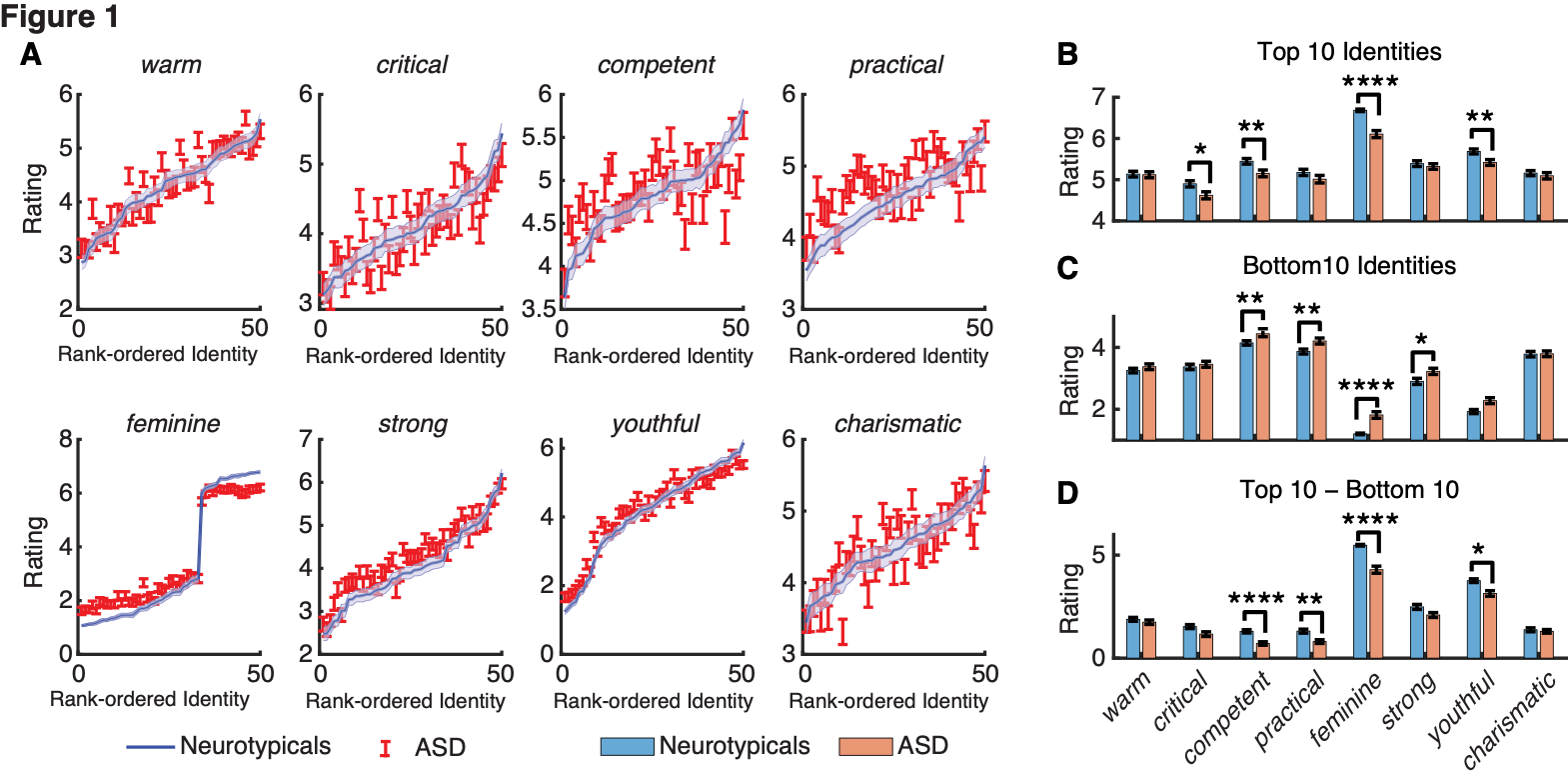


**Figure S1.** Participants with ASD demonstrated reduced specificity in social trait judgments. **(A)** Ratings for each face identity rank-ordered by mean ratings from neurotypicals. Red: ASD. Blue: neurotypicals. Error bars and error shades denote ±SEM across rating modules. **(B)** Average ratings for the 10 identities with the highest ratings from neurotypicals. **(C)** Average ratings for the 10 identities with the lowest ratings from neurotypicals. **(D)** Difference in ratings between the top 10 and bottom 10 identities. Error bars denote ±SEM across rating modules. Asterisks indicate a significant difference between participants with ASD and neurotypicals using two-tailed two-sample *t*-test. *: *p* < 0.05, **: *p* < 0.01, ***: *p* < 0.001, and ****: *p* < 0.0001.


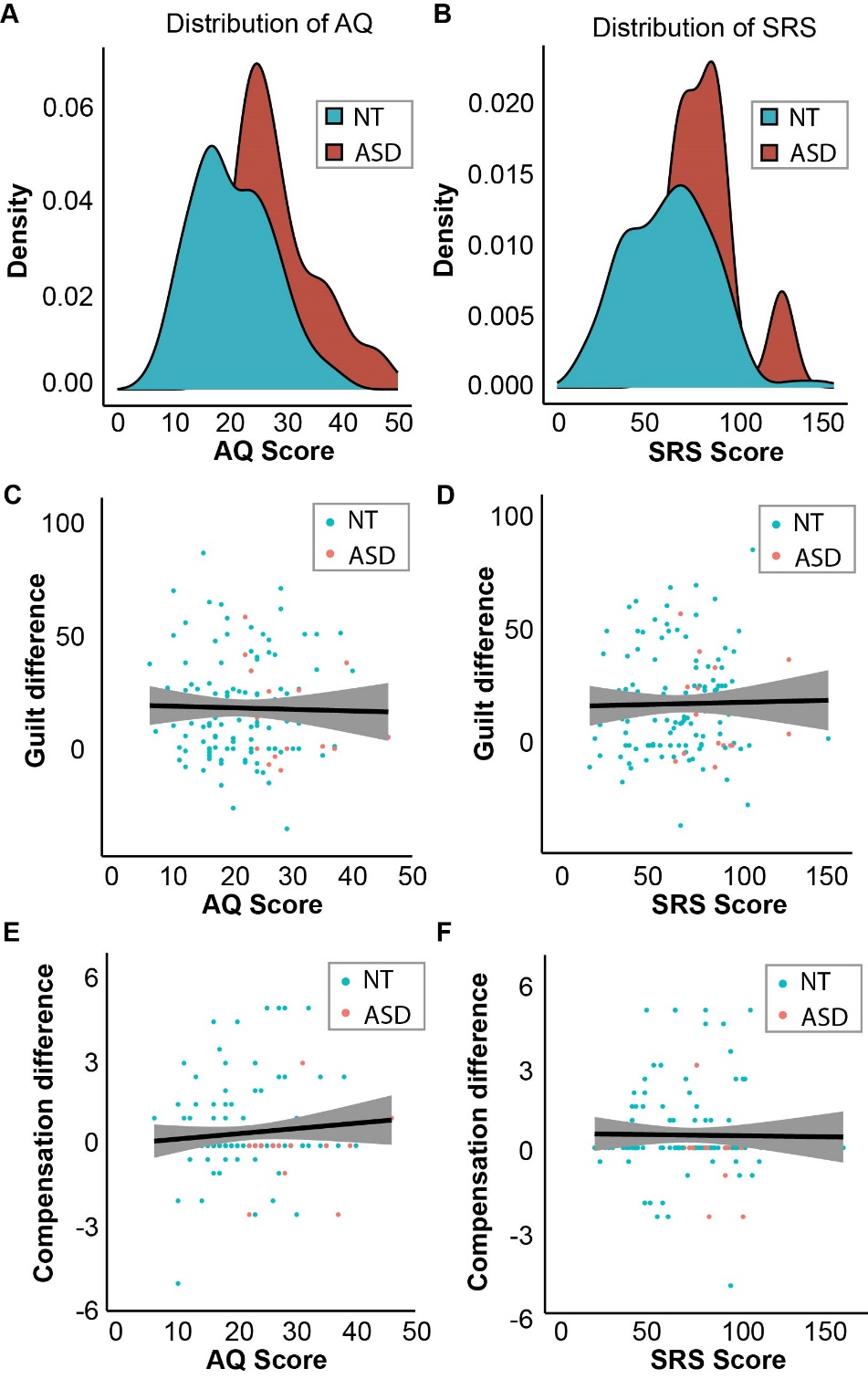


**Figure S2.** Associations between continuous autistic tendencies (AQ and SRS scores) and guilt-related processes. (A-B) Histograms of distribution of AQ and SRS, separately for the neurotypical (NT) and ASD groups. (C-D) Correlations between self-reported guilt difference and autistic tendencies. (E-F) Correlations between compensation tendency difference and autistic tendencies.

*
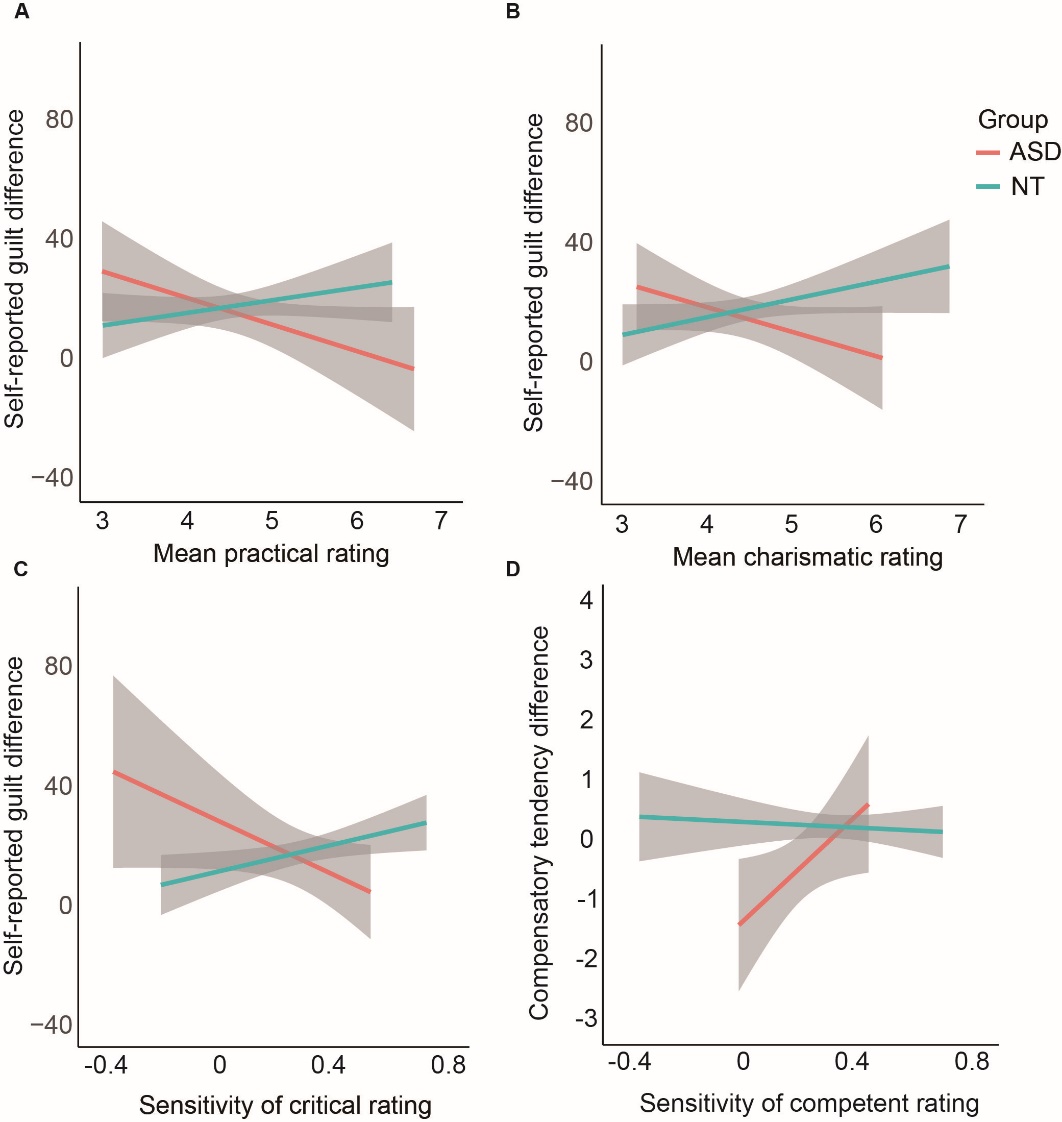
*

**Figure S3**. Associations between guilt-related processes and social trait judgments. (A-B) Associations between self-reported guilt difference and central tendency (i.e., participant-level average) of *practical* and *charismatic* judgments. (C-D) Associations between guilt-related processes and sensitivity in *critical* and *competent* judgments. Note that none of these effects survived multiple comparisons correction.

**Supplemental References**

1. Liu, Z., Luo, P., Wang, X. & Tang, X. Deep learning face attributes in the wild. in *Proceedings of the IEEE international conference on computer vision* 3730–3738 (2015).

2. Lin, C., Keles, U. & Adolphs, R. Four dimensions characterize attributions from faces using a representative set of English trait words. *Nature Communications* **12**, 1–15 (2021).

3. Cao, R. *et al.* Comprehensive social trait judgments from faces in autism spectrum disorder. *Psychological Science* **34**, 1121–1145 (2023).

4. Wang, S. & Adolphs, R. Reduced specificity in emotion judgment in people with autism spectrum disorder. *Neuropsychologia* **99**, 286–295 (2017).

5. de Wit, T. C. J., Falck-Ytter, T. & von Hofsten, C. Young children with Autism Spectrum Disorder look differently at positive versus negative emotional faces. *Research in Autism Spectrum Disorders* **2**, 651–659 (2008).

6. Pelphrey, K. A. *et al.* Visual scanning of faces in autism. *Journal of autism and developmental disorders* **32**, 249–261 (2002).

7. Wang, S. *et al.* Atypical visual saliency in autism spectrum disorder quantified through model-based eye tracking. *Neuron* **88**, 604–616 (2015).
